# Sitagliptin: review of preclinical and clinical data regarding incidence of pancreatitis

**DOI:** 10.1111/j.1742-1241.2010.02382.x

**Published:** 2010-06

**Authors:** S S Engel, D E Williams-Herman, G T Golm, R J Clay, S V Machotka, K D Kaufman, B J Goldstein

**Affiliations:** Merck Research LaboratoriesRahway, NJ, USA

## Abstract

Recent case reports of acute pancreatitis in patients with type 2 diabetes (T2DM) treated with incretin-based therapies have triggered interest regarding the possibility of a mechanism-based association between pancreatitis and glucagon-like peptide-1 mimetics or dipeptidyl peptidase-4 (DPP-4) inhibitors. The objective of this review was to describe the controlled preclinical and clinical trial data regarding the incidence of pancreatitis with sitagliptin, the first DPP-4 inhibitor approved for use in patients with T2DM. Tissue samples from multiple animal species treated with sitagliptin for up to 2 years at plasma exposures substantially in excess of human exposure were evaluated to determine whether any potential gross or histomorphological changes suggestive of pancreatitis occurred. Sections were prepared by routine methods, stained with haematoxylin and eosin and examined microscopically. A pooled analysis of 19 controlled clinical trials, comprising 10,246 patients with T2DM treated for up to 2 years, was performed using patient-level data from each study for the evaluation of clinical and laboratory adverse events. Adverse events were encoded using the Medical Dictionary for Regulatory Activities (MedDRA) version 12.0 system. Incidences of adverse events were adjusted for patient exposure. Tissue samples from preclinical studies in multiple animal species did not reveal any evidence of treatment-related pancreatitis. The pooled analysis of controlled clinical trials revealed similar incidence rates of pancreatitis in patients treated with sitagliptin compared with those not treated with sitagliptin (0.08 events per 100 patient-years vs. 0.10 events per 100 patient-years, respectively). Preclinical and clinical trial data with sitagliptin to date do not indicate an increased risk of pancreatitis in patients with T2DM treated with sitagliptin.

**Review Criteria**

An overview of the literature was performed to describe the prevalence and aetiology of pancreatitis. The effect of sitagliptin on pancreatic histology was evaluated in different species including mice, rats, dogs and monkeys. The incidence of pancreatitis with sitagliptin was analysed by pooling data from 19 controlled clinical trials with sitagliptin.

**Message for the Clinic**

The incidence of pancreatitis is increased in patients with type 2 diabetes (T2DM), and cases of pancreatitis have been reported in patients using most categories of antihyperglycemic medications. Recent postmarketing reports of pancreatitis in patients using incretin-based antihyperglycemic medications [i.e. the glucagon-like peptide-1 receptor (GLP-1R) agonist, exenatide and the dipeptidyl peptidase-4 (DPP-4) inhibitor, sitagliptin] have focused attention on this issue. Review of available preclinical and controlled clinical trial data do not indicate an increased risk of pancreatitis in patients treated with the DPP-4 inhibitor sitagliptin.

## Introduction

Over the last decade, stimulation of glucagon-like peptide-1 (GLP-1) receptor-mediated signalling has been well validated as an approach for the treatment of type 2 diabetes (T2DM). The GLP-1 receptor agonists, GLP-1(7-36)-amide and GLP-1(7-37), hereafter collectively referred to as GLP-1, are produced and secreted from enteroendocrine L-cells of the intestinal epithelium. Key mechanisms responsible for glucose lowering by GLP-1 receptor agonism are stimulation of glucose-dependent insulin biosynthesis and secretion, inhibition of glucagon release and delayed gastric emptying.

Glucagon-like peptide-1 is rapidly hydrolysed *in vivo* (*t*_1/2_ ∼ 1–2 min) to produce a non-insulinotropic product, GLP-1(9-36) amide or GLP-1(9-37) (1). Dipeptidyl peptidase-4 (DPP-4), a serine dipeptidyl aminopeptidase that cleaves two N-terminal amino acids from GLP-1 to generate a non-insulinotropic peptide with no agonist activity against the GLP-1 receptor, is primarily responsible for this degradation. Because of the rapid proteolysis of GLP-1 by DPP-4, the native peptide is not suitable for therapeutic use. To overcome this problem, DPP-4-resistant GLP-1 receptor agonists were developed as injectable peptides for use in the treatment of T2DM. Exenatide (exendin-4), a GLP-1 mimetic discovered in lizard venom, was the first of these peptides approved for therapeutic use (2).

Pharmacological inhibition of DPP-4 is an alternate approach to increase the circulating concentrations of endogenous active GLP-1 (3). Multiple DPP-4 inhibitors have been identified and shown to stabilise endogenous active GLP-1 and improve glycaemic control in patients with T2DM. In addition to cleavage of GLP-1, DPP-4 has been shown to cleave multiple substrates *in vitro*, but few of these substrates have been validated as physiological substrates in humans. GLP-1 and another incretin, glucose-dependent insulinotropic polypeptide (GIP), are well-validated incretin substrates in humans, and both are rapidly metabolised to inactive peptides by the action of DPP-4. In mice, both GLP-1 and GIP have been shown to mediate the acute glucose lowering effects of DPP-4 inhibitor treatment in a glucose challenge paradigm (4). In patients with T2DM, however, because the insulinotropic effect of GIP may be diminished in this disease, DPP-4 inhibitors are believed to mediate glucose lowering primarily via stabilisation of GLP-1 (5).

Interest in the relationship between antihyperglycaemic agents (AHAs) and pancreatitis has recently emerged, triggered originally by reports of acute pancreatitis in patients with T2DM treated with exenatide (6,7). Initially described in a case report in 2006, subsequent postmarketing reports of acute pancreatitis in patients treated with exenatide as well as in patients treated with the DPP-4 inhibitor sitagliptin (8), the first DPP-4 inhibitor approved for use in patients with T2DM, have led to a focus on both the preclinical and clinical experiences with exenatide, other members of the GLP-1 agonist class, and the DPP-4 inhibitor class. In this review, we discuss the association of pancreatitis with T2DM, potential relationships between pancreatitis and medications other than sitagliptin used to treat patients with T2DM, and preclinical and clinical data on the incidence of pancreatitis in patients treated with sitagliptin.

## Aetiology and epidemiology of pancreatitis in type 2 diabetes mellitus

The aetiologies of pancreatitis have been well described in numerous population studies (9). The most common inciting factors are gallstones (35–40%) and alcohol abuse (∼30%) (10). Other risk factors for the development of acute pancreatitis include anatomic abnormalities, hypertriglyceridaemia, obesity, advancing age and use of drugs associated with pancreatitis. Patients with T2DM, who have a higher incidence of several of these known risk factors, have also been shown to have a higher incidence of pancreatitis relative to the general population. For example, in a multinational, placebo-controlled clinical trial involving nearly 10,000 patients with T2DM, the incidence of pancreatitis in the placebo group was 23 out of 4900 patients, or 0.47%, over 5 years (11), for an estimated incidence rate of 0.094 per 100 patient-years. In comparison, annual incidence rates of pancreatitis in the general population have been reported to range from 0.004 to 0.045 per 100 patient-years (12). A recently published study using retrospective claims data from the Ingenix® database, a large commercial US health plan, assessed the incidence of acute pancreatitis in a cohort of patients with T2DM; the reported incidence rate of 0.422 cases per 100 patient-years was greater than the rate of 0.149 cases per 100 patient-years observed in a cohort of general medical patients without diabetes [relative risk = 2.83 (95% CI: 2.61, 3.06)] (13). The rate of pancreatitis increased with age in the non-diabetes cohort, but remained relatively constant with advancing age in patients with T2DM. The reason(s) for the apparent higher risk of pancreatitis in patients with diabetes remains unclear, but may relate to the higher rates of known risk factors for pancreatitis, such as obesity, hypertriglyceridaemia, age and the greater use of medications potentially associated with pancreatitis in patients with T2DM.

## Drug-induced pancreatitis

Drug-induced pancreatitis appears to be a relatively uncommon cause of pancreatitis, although the actual incidence is difficult to determine (14). The use of over 500 medications has been reported in patients with pancreatitis in the literature as anecdotal case reports, although the causal relationship of these medications to cases of pancreatitis remains unclear. This is due, in part, to incomplete information in the case reports regarding dose, time course of onset of pancreatitis in relation to initiation of the suspect medication, other confounding potential aetiologies and variable rechallenge experience. The interpretation of the aetiology of drug-induced pancreatitis in patients with T2DM may frequently be confounded by the concomitant use of medications that are commonly used in these patients and that have been associated with reports of pancreatitis, including statins and angiotensin-converting enzyme inhibitors (15).

Among published case reports of potential drug-induced pancreatitis, there are few regarding AHAs. Patients taking metformin have been reported to develop pancreatitis, although all but one case report involved an overdose or was in the setting of renal failure (16–19). In a case–control study conducted in Sweden between 1995 and 1998, the use of glyburide among patients with T2DM was associated with acute pancreatitis [adjusted odds ratio of 2.5 (95% CI 1.1–5.9)] (20). The use of other members of the sulphonylurea class in case reports of pancreatitis has included glimepiride and gliclazide (21,22). As described above, case reports of acute pancreatitis in patients treated with exenatide have also been published (6,23,24).

In contrast to the limited number of reports in the literature, a relatively higher number of postmarketing reports of pancreatitis associated with a broad range of AHAs has appeared in various databases. These postmarketing events are reported voluntarily from a population of uncertain size; thus, it is generally not possible to establish reliably the frequency of such events or to establish a causal relationship between a medication and a specific adverse event. The Adverse Event Reporting System (AERS), which replaced the Spontaneous Reporting System (SRS) in October 1997, is a computerised information database designed to support the U.S. Food and Drug Administration’s (FDA) postmarketing safety surveillance program for all approved drug and therapeutic biological products. However, as noted on the FDA web site, ‘AERS data do have limitations. First, there is no certainty that the reported event was actually due to the product. FDA does not require that a causal relationship between a product and event be proven, and reports do not always contain enough detail to properly evaluate an event. Further, FDA does not receive all adverse event reports that occur with a product. Many factors can influence whether or not an event will be reported, such as the time a product has been marketed and publicity about an event’ (25). In particular, changes in reporting rates over time because of external factors (e.g. heightened interest in a specific adverse event related to reports of similar events with other medications or increased reporting rates for newly introduced medications) have been identified as significant factors that confound comparisons between medications regarding postmarketing reports of adverse events (26). Thus, it is generally understood that ‘AERS cannot be used to calculate the incidence of an adverse event in the U.S. population’ (25).

Despite these limitations, the AERS database provides a method to aggregate submitted postmarketing reports (27), which has resulted in recent updates to the prescribing information regarding postmarketing reports of pancreatitis for both exenatide and sitagliptin. In the context of the heightened interest regarding the association of GLP-1 receptor agonists and DPP-4 inhibitors with pancreatitis, a search of the AERS and SRS databases for reports of pancreatitis observed with other classes of AHAs was conducted by the authors, using data from 1968 through the third quarter of 2008. This analysis revealed reports of pancreatitis in patients using acarbose, chlorpropamide, exenatide, glimepiride, glipizide, insulin, metformin, miglitol, nateglinide, pioglitazone, pramlintide, repaglinide and rosiglitazone. A search of the same databases for commonly used AHAs (not including insulin), using data from 1968 through the first quarter of 2009, revealed cases of severe pancreatitis (i.e. haemorrhagic or necrotising) in which the following drugs were considered suspect therapy: acarbose, metformin, glimepiride, repaglinide, pioglitazone and rosiglitazone, in addition to exenatide and sitagliptin. Thus, pancreatitis in patients receiving AHAs in the postmarketing environment has been reported across a broad range of mechanistic categories and across the entire range of clinical severity. However, whether these reports are truly reflective of a relationship between the medications and the development of pancreatitis, or simply reflective of the increased rate of pancreatitis in the population of patients with T2DM, remains undetermined.

Pharmacoepidemiological studies can also be used to assess the incidence of postmarketing adverse events through the use of insurance or health system databases that comprehensively capture diagnostic and prescription information. In one such study, Dore et al. reported that the rates of acute pancreatitis among exenatide- or sitagliptin-treated patients were similar to those observed among metformin- or glyburide-treated patients (28). Similarly, Herrera et al. described similar rates of acute pancreatitis among patients prescribed exenatide, sitagliptin or other oral AHA therapies (29). While such data are reassuring, retrospective pharmacoepidemiological studies can be confounded by other factors (30). For example, interpretation of such analyses can be limited by the preferential channelling of patients to specific therapies, which can lead to a bias that cannot easily be adjusted for when interpreting results (31,32). Thus, controlled trials provide the most rigorous method for assessing the incidence of adverse effects of treatments.

## Preclinical studies of sitagliptin

Extensive preclinical toxicity studies were performed as part of the sitagliptin development programme that informs on the occurrence of pancreatitis in a range of animal species: in rats, separate 2-week, 3-month, 6-month and 2-year studies comprising approximately 600 rats exposed to sitagliptin; in mice, separate 3-month and 2-year studies comprising approximately 550 mice exposed to sitagliptin; in dogs, 2-week, 3-month, 6-month and 1-year studies comprising 96 dogs exposed to sitagliptin as well as a 3-month study comprising 45 dogs exposed to the combination of sitagliptin and metformin; and in monkeys, a 3-month study comprising 24 cynomolgus monkeys exposed to sitagliptin (33).

In these studies in non-diabetic animals, sections from the pancreas were reviewed for potential pancreatic toxicity. In all species studied, the pancreas was carefully evaluated to determine whether any potential gross or histomorphological changes associated with administration of sitagliptin occurred. Sections were prepared by routine methods, stained with haematoxylin and eosin, and examined microscopically. Oral administration of sitagliptin for 3 months in monkeys, up to 12 months in dogs and up to 2 years in rats and mice was not associated with gross or histomorphological changes in the pancreas. There was no evidence of drug treatment-related acute pancreatitis in any species studied.

These preclinical toxicity studies were performed with doses that provided plasma exposures in excess of anticipated human exposures (based on the recommended dose of sitagliptin 100 mg/day), as measured by the 24-h area under the plasma concentration time curve (AUC_0–24_). In the above studies, the highest dose tested in a 3-month study in rats was 2000 mg/kg/day, providing approximately a 271-fold margin over human exposure. In a 6-month study in rats, the highest dose studied was 180 mg/kg/day, providing approximately a 23-fold margin over human exposure. In the 2-year rat and mouse studies, the highest dose studied was 500 mg/kg/day, providing approximately a 56- and 68-fold margin, respectively, over human exposure. In dogs, the highest dose studied was 50 mg/kg/day, providing approximately a 28-fold margin over human exposure. In monkeys, the highest dose studied was 100 mg/kg/day, providing approximately a 28-fold margin over human exposure. Thus, at exposures well in excess of the expected human exposure, these preclinical studies did not reveal any evidence that administration of high doses of sitagliptin results in changes in the pancreas of non-diabetic rats, mice, dogs or monkeys.

A recent publication by Matveyenko et al. reported studies in which sitagliptin and metformin were administered orally to transgenic rats overexpressing human islet amyloid polypeptide (HIP) in the pancreas, a potential model of human T2DM (34). In one of these studies, 2-month old wild-type and HIP rats were fed a high-fat diet (HFD) and assigned to one of five groups (*n* = 7–9); wild-type (no drug), HIP rats (no drug), HIP rats administered sitagliptin (200 mg/kg/day), HIP rats administered metformin (200 mg/kg/day) and HIP rats administered sitagliptin (200 mg/kg/day) + metformin (200 mg/kg/day). Exposure levels in the HIP rats following a dose of 200 mg/kg/day of sitagliptin were not reported in this study but, based on previous data, this dose is likely to have produced exposures approximately 20-fold above exposures likely to occur in humans administered the recommended dose of sitagliptin 100 mg/day. Sitagliptin and metformin were administered orally for 12 weeks. In this study, upon histomorphological evaluation of the pancreas from these transgenic animals, it was noted that one of the 16 animals treated with sitagliptin, with or without metformin, had an area of pancreatitis. This area showed marked necrotising pancreatitis characterised by haemorrhagic necrosis, fibrosis, inflammatory cell infiltration and areas of ductal metaplasia. The authors stated that there were no observed effects in any HIP rats not treated with sitagliptin, and that pancreatitis was not observed in any of the other 89 HIP rats evaluated previously. However, the interpretation of this isolated finding is complicated by the limited amount of appropriate control data. The historical data referenced in the paper appears to include only approximately 13 HIP rats that were placed on HFD to induce insulin resistance and hyperglycaemia. Thus, in the historical ‘control’ database, the limited number of animals fed a HFD may have influenced the incidence of pancreatitis.

In contrast to the above findings of Matveyenko et al. (34), using the high-fat/streptozotocin murine model for T2DM in studies conducted at Merck Research Laboratories (35), no pancreatic histopathological effects were observed with sitagliptin treatment (33). To generate this model, 4-week-old male ICR mice were placed on a HFD in which 60% of energy intake is from fat. After 3 weeks of HFD, the mice are injected once with low-dose streptozotocin (90–100 mg/kg i.p.) to induce partial insulin deficiency. Three weeks after streptozotocin injection, the majority of HFD/streptozotocin-treated mice display hyperglycaemia, insulin resistance and glucose intolerance. The original purpose of this study was to explore the effects of sitagliptin on beta cell mass and function, and the primary results of the study have been recently published (36). In this study, fifty 10-week old mice were treated with sitagliptin at doses of up to 840 mg/kg/day for up to 10 weeks, resulting in estimated exposures (based on exposure data in CD-1 mice from a 14-week dose-range-finding study conducted to support the development of sitagliptin) as high as approximately 120-fold relative to the exposure in humans administered the recommended dose of 100 mg/day. Background changes of very slight focal chronic inflammation were seen in the pancreas in both control (*N* = 41) and streptozotocin-treated (*N* = 41) animals at similar incidences, with no difference noted in sitagliptin-treated animals (33). Of additional note is the study by Koehler et al., in which the effect of sitagliptin on the expression of genes associated with the development of pancreatitis in mice was compared with metformin and the GLP-1 receptor agonists exenatide and liraglutide (37). In contrast to the GLP-1 receptor agonists, neither sitagliptin nor metformin significantly altered pancreatic gene expression profiles. The same laboratory reported that in C57BL/6 mice (*N* = 6) treated with sitagliptin at doses as high as ∼370 mg/kg/day, no histological evidence of pancreatitis was noted (D. Drucker, personal communication, University of Toronto, Toronto, ON, Canada). Another recent report described an increase in pancreatic acinar inflammation in Sprague–Dawley rats after chronic administration of exenatide 10 μg/kg (38), although the potential mechanism(s) responsible for this finding in this rat model remains speculative.

Thus, with the exception of a report of the histological findings in a single animal from a study of a genetically-altered rat model of diabetes, a broad range of preclinical studies in both non-diabetic and diabetic animals at exposures exceeding human exposure did not demonstrate a relationship between use of sitagliptin and the development of pancreatitis.

## Clinical experience with sitagliptin

A previously published, pooled analysis of data from 12 double-blind, randomised clinical studies of up to 2 years in duration in patients with T2DM, comprising 6139 patients treated with either sitagliptin or a comparator agent (placebo or other AHA), was conducted to assess for differences in the incidence of adverse events between patients treated with sitagliptin and patients not exposed to sitagliptin (39). This pooled population included patients treated with the usual clinical dose of sitagliptin 100 mg/day (administered either as 100 mg q.d. or 50 mg b.i.d.) or concurrent control for between 12 and 106 weeks in clinical studies that were complete as of November 2007. Patients in the sitagliptin group (*N* = 3415) received sitagliptin when used as monotherapy, initial combination therapy with metformin, or add-on combination therapy with other AHAs including metformin, pioglitazone, a sulphonylurea (with and without metformin), or metformin + rosiglitazone. Patients in the control (non-exposed) group (*N* = 2724) received placebo, pioglitazone, metformin, a sulphonylurea (with and without metformin), or metformin + rosiglitazone. From each contributing study, the pooling was conducted by including portions of studies with controlled, parallel treatment groups. In this pooled analysis, no difference in the incidence of pancreatitis between patients treated with sitagliptin and patients not exposed to sitagliptin was observed (39).

To examine more comprehensively the safety and tolerability of sitagliptin, an updated pooled analysis of data from 19 double-blind, randomised clinical studies (including 7 additional studies relative to the prior pooled analysis) of up to 2 years in duration in patients with T2DM that were complete as of July 2009, and comprising 10,246 patients treated with either sitagliptin or a comparator agent (placebo or other AHA), was recently completed. Patients in the sitagliptin group (*N* = 5429) received sitagliptin (as either 100 mg q.d. or 50 mg b.i.d.) when used as monotherapy, initial combination therapy with either metformin or pioglitazone, or add-on combination therapy with other AHAs including metformin, pioglitazone, a sulphonylurea (with and without metformin), insulin (with and without metformin), or metformin + rosiglitazone. Patients in the non-exposed group (*N* = 4817) received placebo, pioglitazone, metformin, a sulphonylurea (with and without metformin), insulin (with and without metformin), or metformin + rosiglitazone. As in the prior pooled analysis, from each contributing study, the pooling was conducted by including portions of studies with controlled, parallel treatment groups.

This safety analysis used patient-level data from each study for the evaluation of clinical and laboratory adverse events. Adverse events were encoded using the MedDRA (Medical Dictionary for Regulatory Activities; version 12.0) system, a validated terminology database developed by the International Conference on Harmonisation. The specific MedDRA preferred terms used in this analysis were pancreatitis, acute pancreatitis and chronic pancreatitis. To account for the different exposures for the sitagliptin group compared with the non-exposed group, an exposure-adjusted analysis of incidence was conducted. For patients who had one or more events, person-time was computed beginning with the date of randomisation and ending with the date of the first event. For patients who did not have an event, person-time was computed beginning with the date of randomisation and ending 14 days after the last dose of study medication. Adverse events were expressed as exposure-adjusted incidence rates (i.e. number of patients with an event divided by patient-years of exposure). Differences in incidence rates between treatment groups were computed for all end-points, and the corresponding 95% confidence intervals (CIs) were calculated using the method of Miettinen and Nurminen (40), stratified by study. In most studies included in this analysis, glycaemic rescue therapy was to be implemented based upon protocol-specified hyperglycaemic criteria. Glycaemic rescue medications included metformin, pioglitazone, a sulphonylurea, or increased doses of insulin (in the add-on to insulin study). The analysis in this pooled safety population focused on the results that included data obtained both before and after a patient initiated rescue therapy.

As presented in Table 1, the incidence rate for the combined adverse events of pancreatitis and pancreatitis acute was similar for both groups (0.08 and 0.10 per 100 patient-years), with a between-group difference (95% CI) of −0.02 (−0.20, 0.14). For the specific events of ‘pancreatitis acute’ and ‘pancreatitis’, the 95% CI for the between-group difference in the event rates also included zero. For the adverse event of chronic pancreatitis, the event rates per 100 patient-years were 0.04 and 0.03 for the sitagliptin group and the non-sitagliptin-exposed group, respectively, with a between-group difference (95% CI) of 0.02 (−0.11, 0.13). In these clinical trials, there were no cases of haemorrhagic or necrotising pancreatitis, and no fatalities associated with pancreatitis were reported. Among the four patients in the sitagliptin group who had an adverse event of pancreatitis or pancreatitis acute, one had a prior medical history of recurrent pancreatitis, two had pancreatitis associated with gallstones and one had severe hypertriglyceridaemia. Among the four patients in the non-exposed group who had an adverse event of pancreatitis or pancreatitis acute, two had a prior medical history of chronic pancreatitis. Thus, this recent pooled analysis of 19 controlled clinical studies does not suggest an increased risk of pancreatitis in patients treated with sitagliptin.

**Table 1:** Person-time adjusted analysis of pancreatitis and pancreatitis acute adverse events including data after glycaemic rescue: sitagliptin 100 mg vs. non-exposed

Adverse event end-point	Treatment	*n*/Patient-years of exposure (100 patient-years event rate)*	Difference vs. non-exposed (95% CI)†
Pancreatitis/pancreatitis acute	Sitagliptin 100 mg	4/4708 (0.08)	−0.02 (−0.20, 0.14)
	Non-exposed	4/3942 (0.10)	
Pancreatitis	Sitagliptin 100 mg	3/4708 (0.06)	0.06 (−0.04, 0.19)
	Non-exposed	0/3943 (0.00)	
Pancreatitis acute	Sitagliptin 100 mg	1/4709 (0.02)	−0.08 (−0.25, 0.03)
	Non-exposed	4/3942 (0.10)	

*n* = Number of patients with ≥ 1 occurrence of the end-point.

* Patient-years of exposure were computed as the total time in the treatment period + 14 days for patients who did not have an event, and as the total time up to the time of the first event for patients who had an event.

† 95% CI computed using the Miettinen & Nurminen method stratified by study.

## Conclusion

Assessment of the safety of investigational and marketed drugs is an ongoing process that incorporates a variety of distinct, yet complementary, approaches. These approaches include, among others, preclinical studies in multiple species, typically involving drug exposures that greatly exceed the anticipated exposure in patients; controlled Phase I clinical studies in healthy subjects, also typically involving drug exposures that exceed the anticipated exposure in patients; controlled Phase II and Phase III clinical studies in the targeted patient population at therapeutic drug exposures; and postapproval analyses of clinical trial data, spontaneous postmarketing reports of adverse events and pharmacoepidemiological studies of large databases. As described in the present report, the preclinical and clinical trial data developed with sitagliptin to date do not indicate an increased risk of pancreatitis in patients with T2DM treated with sitagliptin. Nevertheless, as postmarketing events of pancreatitis have been reported for patients with diabetes while being treated with various AHAs, including sitagliptin, continued surveillance of the postmarketing experience and assessment of adverse events in patients participating in controlled clinical trials with sitagliptin are ongoing. Additional preclinical and clinical studies that are directed towards a better understanding of the potential relationship between specific medications, diabetes itself, and the incidence and severity of pancreatitis may lead to further knowledge in this area.

## References

[1] Meier JJ, Nauck MA (2005). Glucagon-like peptide 1 (GLP-1) in biology and pathology. Diabetes Metab Res Rev.

[2] Iltz JL, Baker DE, Setter SM (2006). Exenatide: an incretin mimetic for the treatment of type 2 diabetes mellitus. Clin Ther.

[3] Drucker DJ (2007). Dipeptidyl peptidase-4 inhibition and the treatment of type 2 diabetes. Diabetes Care.

[4] Flock G, Baggio LL, Longnet C (2007). Incretin receptors for glucagon-like peptide 1 and glucose-dependent insulinotropic polypeptide are essential for the sustained metabolic actions of vildagliptin in mice. Diabetes.

[5] Nauck MA, Heimesaat MM, Orskov C (1993). Preserved incretin activity of glucagon-like peptide 1 [7-36 amide] but not of synthetic human gastric inhibitory polypeptide in patients with type-2 diabetes mellitus. J Clin Invest.

[6] Denker PS, Dimarco PE (2006). Exenatide (exendin-4)-induced pancreatitis: a case report. Diabetes Care.

[7] Bain SC, Stephens JW (2008). Exenatide and pancreatitis: an update. Expert Opin Drug Saf.

[8] U.S. Food and Drug Administration Information for Health Care Professionals - Acute Pancreatitis and Sitagliptin (marketed as Januvia or Janumet).

[9] Spanier BWM, Dijkgraaf MGW, Bruno MJ (2008). Epidemiology, aetiology and outcome of acute and chronic pancreatitis: an update. Best Pract Res Clin Gastroenterol.

[10] Forsmark CE, Billie J (2007). AGA institute technical review on acute pancreatitis. Gastroenterology.

[11] Keech A, Simes RJ, Barter P (2005). Effects of long-term fenofibrate therapy on cardiovascular events in 9795 people with type 2 diabetes mellitus (the FIELD study): randomised controlled trial. Lancet.

[12] Yadov D, Lowenfels AB (2006). Trends in the epidemiology of the first attack of acute pancreatitis: a systematic review. Pancreas.

[13] Noel RA, Braun DK, Patterson RE, Bloomgren GL (2009). Increased risk of acute pancreatitis and biliary disease observed in patients with type 2 diabetes: a retrospective cohort study. Diabetes Care.

[14] Balani AR, Grendell JH (2008). Drug-induced pancreatitis. Drug Saf.

[15] Badalov NS, Baradarian R, Iswara K (2007). Drug-induced acute pancreatitis: an evidence-based review. Clin Gastroenterol Hepatol.

[16] Ben MH, Thabet H, Zaghdoudi I (2002). Metformin associated acute pancreatitis. Vet Hum Toxicol.

[17] Mallick S (2004). Metformin induced acute pancreatitis precipitated by renal failure. Postgrad Med J.

[18] Fimognari FL, Corsonello A, Pastorell R (2006). Metformin-induced pancreatitis: a possible adverse drug effect during acute renal failure. Diabetes Care.

[19] Infante JM, Bermejo ABP, Gallardo BP (2008). Pancreatitis aguda toxica por metformina sin insuficiencia renal. Med Clin (Barc).

[20] Blomgren KB, Sundström A, Steineck G (2002). Obesity and treatment of diabetes with glyburide may both be risk factors for acute pancreatitis. Diabetes Care.

[21] Duboeuf T, De Widerspach-Thor A, Scotto B (2004). Acute glimepiride-induced pancreatitis. Gastroenterol Clin Biol.

[22] Roblin X, Abinader Y, Baziz A (1992). Acute pancreatitis induced by gliclazide. Gastroenterol Clin Biol.

[23] Ayoub WA, Kumar AA, Naguib HS (2009). Exenatide-induced acute pancreatitis. Endocr Pract.

[24] Tripathy NR, Basha S, Jain R (2008). Exenatide and acute pancreatitis. J Assoc Physicians India.

[25] U.S. Food and Drug Administration Adverse Event Reporting System.

[26] Hartnell NR, Wilson JP (2004). Replication of the Weber effect using post-marketing adverse event reports voluntarily submitted to the United States Food and Drug Administration. Pharmacotherapy.

[27] Wysowski DK, Swartz L (2005). Adverse drug event surveillance and drug withdrawals in the United States, 1969-2002: the importance of reporting suspected reactions. Arch Intern Med.

[28] Dore DD, Seeger JD, Chan KA (2009). Use of a claims-based active drug safety surveillance system to assess the risk of acute pancreatitis with exenatide or sitagliptin compared to metformin or glyburide. Curr Med Res Opin.

[29] Herrera V, Aubert R, Tully L Pancreatitis in patients treated with exenatide or sitagliptin.

[30] MacMahon S, Collins R (2001). Reliable assessment of the effects of treatment on mortality and major morbidity, II: observational studies. Lancet.

[31] Radican L, Rajagopalan S, Mavros P (2009). Differences in baseline characteristics between patients prescribed sitagliptin versus exenatide based on a US electronic medical record database. Diabetologia.

[32] Zhang Q, Rajagopalan S, Mavros P (2009). Baseline characteristics of patients on sitagliptin and other oral antihyperglycemic agents. Diabetes.

[33] Data on File.

[34] Matveyenko AV, Dry S, Cox HJ (2009). Beneficial endocrine but adverse exocrine effects of sitagliptin in the human islet amyloid polypeptide transgenic rat model of type 2 diabetes: interactions with metformin. Diabetes.

[35] Luo J, Quan J, Tsai J (1998). Nongenetic mouse models of non-insulin-dependent diabetes mellitus. Metabolism.

[36] Mu J, Petrov A, Eiermann GJ (2009). Inhibition of DPP-4 with sitagliptin improves glycemic control and restores islet cell mass and function in a rodent model of type 2 diabetes. Eur J Pharm.

[37] Koehler JA, Baggio LL, Lamont BJ (2009). Glucagon-like peptide-1 receptor activation modulates pancreatitis-associated gene expression but does not modify the susceptibility to experimental pancreatitis in mice. Diabetes.

[38] Nachnani JS, Bulchandani DG, Nookala A (2010). Biochemical and histological effects of exendin-4 (exenatide) on the rat pancreas. Diabetologia.

[39] Williams-Herman D, Round E, Swern AS (2008). Safety and tolerability of sitagliptin in patients with type 2 diabetes: a pooled analysis. BMC Endocrine Disorders.

[40] Miettinen O, Nurminen M (1985). Comparative analysis of two rates. Stat Med.

